# Recent Materials Developed for Dispersive Solid Phase Extraction

**DOI:** 10.3390/molecules25214869

**Published:** 2020-10-22

**Authors:** Piotr Ścigalski, Przemysław Kosobucki

**Affiliations:** Department of Food Analysis and Environmental Protection, Faculty of Chemical Technology and Engineering, UTP University of Science and Technology, Seminaryjna 3, 85-326 Bydgoszcz, Poland; p.kosobucki@utp.edu.pl

**Keywords:** dispersive solid phase extraction, novel sorbents, nanomaterials, environmental samples, food samples

## Abstract

Solid phase extraction (SPE) is an analytical procedure developed with the purpose of separating a target analyte from a complex sample matrix prior to quantitative or qualitative determination. The purpose of such treatment is twofold: elimination of matrix constituents that could interfere with the detection process or even damage analytical equipment as well as enriching the analyte in the sample so that it is readily available for detection. Dispersive solid phase extraction (dSPE) is a recent development of the standard SPE technique that is attracting growing attention due to its remarkable simplicity, short extraction time and low requirement for solvent expenditure, accompanied by high effectiveness and wide applicability. This review aims to thoroughly survey recently conducted analytical studies focusing on methods utilizing novel, interesting nanomaterials as dSPE sorbents, as well as known materials that have been only recently successfully applied in dSPE techniques, and evaluate their performance and suitability based on comparison with previously reported analytical procedures.

## 1. Introduction

The constant increase in the quality of life observed in recent years and decades requires corresponding growth of economy and industry, resulting in increased emissions of potentially harmful substances, such as pesticides, pharmaceuticals, heavy metals and more, collectively known as xenobiotics, into the natural environment. As our understanding of these substances and their effect on the environment and human health grows, restrictions are being put in place to control their release, prompting the need to develop suitable measurement methods, allowing monitoring of their presence in the environment and aiding the industry in reducing polluting emissions.

Numerous precise analytical methods have been developed to this end, many of them consisting of a chemical species separation achieved usually through the means of a wide variety of chromatographic techniques, namely gas chromatography (GC) and liquid chromatography (LC), the latter now mostly employed in its high performance iterations such as HPLC (high pressure liquid chromatography) and UPLC (ultrahigh pressure liquid chromatography), as well as electrophoretic techniques, such as capillary zone electrophoresis (CZE). Target quantification can be carried out based on the analyte type and utilizes one or more of many diverse detection techniques, from atomic emission (AES) or absorption spectroscopy (AAS) in the case of metal ions, to mass spectrometry (MS), to ultraviolet (UV), fluorescence (FL) or visual range (Vis) spectrophotometry for organic compound determination.

However accurate and quick a method should be, samples arriving at the laboratory can rarely be analyzed without any preparation. Environmental samples often exhibit complex matrixes containing many particulate and chemical interferents, rendering them unsuitable for immediate analysis [1,2,3]. Nearly every sample needs to be subjected to treatment aimed at separating the analyte from the matrix, allowing for reliable analysis. For more complex samples, this can necessitate the employment of multiple complicated procedures, in which case the sample pre-treatment becomes the bottleneck of the entire analytical process [1,2,4]. As measurement becomes faster and more accurate, the drive to develop effective, simple, selective, rapid, reliable and green clean-up and pre-concentration techniques is as strong as ever.

One of the oldest and most simple pre-concentration methods still in wide use is liquid–liquid extraction (LLE). This technique, however, carries some inherent disadvantages that can make it expensive or impractical in application: it is time-consuming and labor-intensive while also requiring large volumes of organic solvents, often highly toxic themselves [1,2,3].

Solid phase extraction (SPE) enjoys significant advantages over LLE as it largely manages to avoid many of its drawbacks. It requires much lower amounts of solvents, takes less time and is less complex to operate, while providing better effectiveness and selectivity and also being easier to automate and combine with other pre-treatment or analytical procedures. SPE with its modifications and iterations, such as solid phase microextraction (SPME), stir bar sorptive extraction (SBSE), matrix solid phase dispersion (MSPD) or ion-exchange solid phase extraction (IE-SPE), has rightfully become one of the most widely used sample preparation techniques for solid or liquid samples [1,2,3,5].

Dispersive solid phase extraction (dSPE) is an iteration of the SPE technique that has surged in popularity since 2003, when it was reported by Anastassiades et al. as an effective clean-up procedure in pesticide extraction from produce. In this very approachable method, the solid sorbent is applied directly into the volume of a liquid sample solution without the need for prior sample preparation, and the whole procedure relies entirely on shaking and centrifugation. This simple approach ensures high contact surface between sorbent and sample, allowing for the extraction equilibrium to be reached quickly. The resulting method is rapid and proven to be effective, and considering the very low amounts of sorbent and solvent required, it can be viewed as a more environmentally friendly method than standard solid phase extraction [2,5,6].

**Figure 1:**
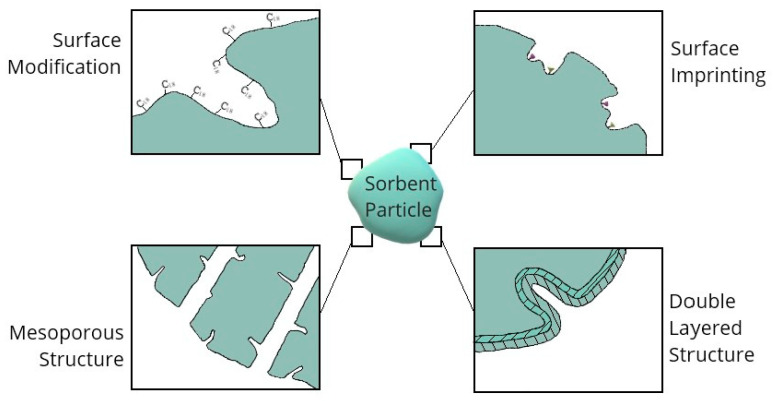
Examples of basic functional structures of SPE sorbents.

dSPE quickly found recognition as a valuable complement to the QuEChERS (quick, easy, cheap, efficient, rugged, safe) clean-up procedure, resulting in the development of CSN EN 15,562 and AOAC 2007.01 official standards for QuEChERS-dSPE pesticide determination methods. Most commonly used commercially available kits developed in accordance with these standards include typically one or more of three sorbents for the dSPE step: graphitized carbon black (GCB), primary-secondary amine (PSA) and octadodecyl-bonded silica (C18). However, new materials are being investigated as suitable for dSPE as the range of perceived potential applications for the technique broadens [2,7]. As shown in Figure 1, materials put to use as sorbents exhibit various functional structures, which can be further modified in order to achieve the highest possible effectiveness and selectivity of the extraction process.

The aim of this review article is to take a closer look at new and interesting materials that have been reported over the last couple years as having potential for application in dispersive solid phase extraction as sorbents. A survey of recently published literature revealed a large interest in exploring the possibilities of dSPE application and improving its efficiency by utilizing newly developed nanomaterials and techniques. In this work, we focused on the most recent reports on novel or modified classical sorbents applied in dSPE procedures and subjected the proposed analytical methods to critical evaluation based on the obtained results and validation parameters.

## 2. Sorbents Based on Silica

Silica in various forms is one of the oldest and most widely used sorbents in solid phase extraction, typically used in normal phase procedures based on adsorption [1]. Its drawbacks include its limited pH tolerance and singular sorption mechanism, stemming from a lack of diverse surface functional groups. It is, however, an easy to synthesize, cheap and rigid material, with good resistance to shrinking and swelling, and the ease with which it can be modified offsets its disadvantages to a significant degree. In addition to this, mesoporous silica adds the benefit of having an organized, ordered internal structure containing uniformly distributed mesopores [8,9,10,11]. Recent attempts to take advantage of the ease with which standard silica can be modified have provided unclear results. Papers describe the immobilization of complex amine-based organic moieties on the surface of the silica carrier and the application of obtained sorbents in the dSPE procedure for the extraction of rare earth elements [12] and phosphoproteins [13]. While the materials show desirable qualities such as high selectivity toward analyte or good reusability, it is difficult to properly evaluate the potential of the proposed methods with no precise numerical data presented. In contrast, a study by Fuh et al. on silanized silica modified with a lauryl methacrylate-based copolymer, used as a sorbent in the dSPE-HPLC-UVD process for the determination of trace herbicide content [14], presented good results, closely comparable to literature data (Table 1). An additional advantage of the method is the remarkably low sample size required; however, the overall precision may suffer from the distinctively limited linear range.

Mesoporous silica has been recognized for carrying similar potential, as a number of commercially available materials, such as MCM (Mobil Composition of Matter), HMS (Hexagonal Mesoporous Silica) and MSN (Mesoporous Silica Nanoparticle), have been modified with quaternary ammonium salts and amines. The obtained sorbents were used in analytical procedures to detect endocrine disrupting compounds (EDC) [15], polyphenols [9,16], as well as synthetic dyes in various food and environmental samples. Results of these studies, gathered and displayed in Table 1, were in all cases comparable or better than cited literature data, while also benefitting from the advantages of dSPE pre-treatment and employing relatively common detection systems, such as HPLC devices coupled with MS or diode array detectors (DAD), proving the high applicability of the proposed methods as well as the rich potential of mesoporous silica as a base for a dSPE sorbent. 

A study that seems to fully embrace this potential focused on applying mesoporous silica SBA-15 modified with metformin to isolate heavy metal ions from food samples [10,17]. When compared to literature data, the proposed method exhibited results close to those achieved using a much more expensive inductively coupled plasma–mass spectrometry (ICP-MS) analysis, confirming its very good efficiency. Great effectiveness of the sorbent itself was proven, with calculated enrichment factor (EF) reaching values as extraordinarily high as 1800. The greatest disadvantage of the method appears to be the more complex operating procedures when compared to other dSPE techniques, as it introduces an additional clean-up step of either surfactant-assisted dispersive liquid–liquid microextraction (SA-dLLME) or ultrasound-assisted emulsification microextraction (UA-EME), as well as a relatively complicated sorbent preparation process.

An equally complex material, combining the characteristics of ionic liquids and mesoporous silica, was the subject of an interesting study focused on the determination of plant growth regulators (PGR) in a herb widely used in traditional Chinese medicine [8]. Laborious sorbent preparation appears to be the greatest issue with the method; however, the operating procedure is similar to the standard dSPE technique and enjoys its benefits, and it obtained results exceeding any data used for reference.

## 3. Sorbents Based on Magnetic Nanoparticles

In recent years, the application of magnetic nanoparticles (MNPs) as sorbents in dSPE procedures has drawn much attention. These materials exhibit good dispersibility and high surface area to volume ratio, and their super-paramagnetic nature makes them easy to isolate from the matrix by an external magnetic field, without retaining residual magnetization. Because of its physicochemical properties, magnetite (Fe_3_O_4_, iron oxide) is the most widely used in dSPE. In order to protect the material from oxidation and prevent the formation of agglomerates, it is often coated with organic or inorganic coatings, which in turn opens up the option of MNP modification and functionalization [18,19,20].

The majority of recent studies on MNP-based sorbents reported their extraction methods to be highly accurate, with recovery values close to 100% (Table 2). The most notable exception was described in a series of papers by Hernández-Borges et al., presenting the results of studies on magnetite coated with poly(dopamine) (Fe_3_O_4_@pDA), applied as a dSPE sorbent for the determination of phthalic acid esters (PAE) in sand and water, as well as mycotoxins in dairy product samples [20,21,22]. Despite the fairly low precision of the extraction process, the employed analytical procedures were highly sensitive, with obtained values comparable to or better than reported in any cited reference.

The highest extraction accuracy with MNP-based sorbent was reported in a study on the determination of arsenic speciation analysis in water, using poly(methacrylic acid) (PMAA) coated magnetite particles [23]. The sorbent, denoted as M-PMA, proved incredibly stable, showing no significant loss of sorption capabilities after up to 25 sorption–desorption cycles. The sensitivity of the method, employing a hybrid generation microwave plasma atomic emission spectrometry (HG-MP-AES) detection system, marginally falls behind only the most effective analytical procedures. While this is the case for most of the recently reported analytical procedures making use of MNP-based dSPE sorbents, a method for trihalomethane (THM) determination [24] proposed by Farhadi et al. yielded unremarkable results. Here, magnetite nanoparticles were coated with zein, a protein found in maize, which allowed very accurate extraction when applied as a sorbent. Using headspace thermal desorption (HSTD) followed by GC coupled with a micro electron capture detection (MECD) system, however, provided THM detection with sensitivity noticeably lower than in referential literature.

## 4. Sorbents Based on Molecularly Imprinted Polymers

Molecularly imprinted polymers (MIPs) have received broad recognition and found widespread application in residue detection techniques, considered one of the most selective phases applied in SPE methods. Their preparation includes a copolymerization reaction of a functional monomer, capable of interacting with the target analyte, with a crosslinker in the presence of a template, that is either the target analyte or its close derivative. After the reaction, template molecules are removed from the structure, leaving a site on the surface that is complementary in size, shape and chemical functionality to the target analyte. This ensures very high specificity and selectivity of MIPs, sought after in extraction procedures, and prompts their denomination as synthetic antibodies [1,2,28,29].

MIP materials recently applied as sorbents in dSPE procedures generally fulfil the expectation of high selectivity, whether the target analytes are metal ions [19,30,31], polyaromatic hydrocarbons (PAHs) [28] or cephalosporin antibiotics [32]; however, other parameters of these analyses varied (Table 3). Good selectivity was also confirmed for a methacrylic acid (MAA)–ethylene glycol dimethacrylate (EGDMA) copolymer sorbent with dual templates (dt-MIP), designed for the simultaneous separation of two different fluoroquinolones (FQ), norfloxacin (NOR) and enrofloxacin (ENR) [33], as the sorbent showed much higher affinity toward targets than their structural analogues. Utilizing the dSPE technique with the HPLC-DAD system allowed us to carry out analyte determination with parameters better or comparable to the cited reference; however, the sorbent itself exhibited relatively low sorption capacity.

Out of the proposed MIP dSPE procedures, there was one sorbent that did not meet the selectivity expectation. This was reported by Wang et al., who synthesized hydrophilic molecularly imprinted microspheres (HMIMs) using hydroxypropyl methacrylate as a functional monomer and EGDMA as crosslinker in the presence of azoxystrobin fungicide as target template [29]. HMIM selectivity was investigated by carrying out extraction from a mixed solution of three fungicides, including azoxystrobin, which was absorbed in only marginally higher amounts. Moreover, a similar test was performed using a non-imprinted polymer and the obtained results were also comparable. It is worth noting, however, that in a procedure utilizing LC-UV detection, HMIM allowed for target determination in vegetables with good sensitivity within a very wide concentration range.

## 5. Sorbents Based on Carbon

Due to its large surface area, high adsorption capacity as well as outstanding chemical, mechanical and thermal stability and relatively low preparation cost, carbon in different forms has seen wide application in xenobiotic removal from the environment. As these properties are valued for materials used in separation techniques, it is only natural for different carbon allotropes to attract considerable attention as potential SPE sorbents. In fact, as mentioned earlier, graphitized carbon black is one of the sorbents currently utilized and distributed with the QuEChERS standard procedure kits as a sorbent for the dSPE clean-up step [6,35,36,37].

Carbon forms investigated as sorbents range from allotropes, such as carbon nanotubes (CNTs), graphene and graphite, to modified carbon structures, known as activated carbon (AC). The latter can be produced through thermal and chemical activation of either natural or synthetic material with high carbon content within its structure, including some types of waste—for instance, waste tires [35]. Biomaterials are also being investigated as potential precursors for AC, and these include almond and walnut shells, orange peels and others. The type of precursor used, as well as the applied activation method, greatly impacts the physicochemical properties and characteristics of the obtained material [35,38]. To this end, Ebrahimi et al. reported a study on an AC material based on cherry stones [38] that was applied in the dSPE of copper followed by flame atomic absorption spectrometry (FAAS) detection. The activation process included both chemical and thermal steps, and to further improve the material’s sorption capacity, it was subjected to acidic and microwave modifications. Extraction efficiency, illustrated with enrichment factor EF reaching a value of 100, proved to be better than in any of the reported SPE studies used as a reference, as was the overall method sensitivity, displayed in Table 4. The method’s applicability was evaluated by carrying out the extraction from spiked milk, macaroni and rice samples, which yielded highly accurate recoveries, and the acid and microwave modified activated carbon (AMM-AC) sorbent, exhibited satisfactory reusability, showing no significant decrease in effectiveness for up to six sorption cycles. The proposed method appears to be a strong competitor for copper determination; however, it has a potentially severe drawback, as the selectivity test revealed the sorbent’s low tolerance for the presence of Co^2+^, No^2+^ and Pd^2+^ ions in the matrix, which needs to be taken into account. A study investigating the applicability of chemically activated, tire waste-originated AC deposited on the surface of polyacrylonitrile (PAN) nanofibers as a dSPE sorbent [35] shows its good ability to extract fluoroquinolones, allowing for high detection sensitivity when paired with the HPLC-DAD system. The method yielded excellent recovery values and the reusability test showed that the material can be utilized efficiently for up to ten times, after which threshold a sharp decline in sorptive properties is observed.

### 5.1. Graphite and Graphene

Graphite is a natural, crystalline carbon allotrope with a hexagonal internal structure. It found wide use in chromatography and solid phase extraction as it exhibits a large surface area and therefore high adsorption capacity, has good mechanical, electric and thermal resistance and conductivity and appears to be nearly inert chemically. These remarkable properties, however, are amplified in recently discovered material initially derived from graphite, graphene, which could be described as a single layer of graphite, an essentially two-dimensional sheet of hexagonally arranged carbon atoms that is just one atom thick. As the carbon rings forming the structure exhibit aromatic properties, it possesses a large delocalized π-electron system, making it possible to enter strong interactions with other aromatic compounds. Additionally, graphene derivatives, such as graphene oxide (GO), can be readily modified to employ additional functionalization to further adjust the sorption performance of the material [39,40].

As graphene has a tendency for irreversible aggregation that diminishes its sorptive properties, studies have been carried out to alleviate this problem through its immobilization or modification [36,39,40,41,42,43]. Although the provided literature reference was very limited, remarkable sensitivity and good accuracy were obtained when applied to real samples, as well as good sorbent reusability, attesting to the high potential of the proposed methods and sorbents (Table 4). An interesting method proposed by El-Wekil et al. saw the use of cobalt hydroxide nanoparticles (CHNPs) as sorbents complementary to reduced graphene oxide (rGO) in the dSPE procedure [44]. CHNP addition inhibited graphene aggregation and therefore ensured the high surface area of the sorbent, as well as decreasing its binding affinity towards the surfactant, polyethylene glycol (PEG) 6000, used in a cloud point extraction (CPE) system, via the formation of rGO-CHNPs hydrogen bonds. This method was proven to be highly tolerant towards potentially interfering compounds and ions and the calculated sensitivity and accuracy placed it among the most effective methods cited from the literature. In addition, the sorbent reusability test showed no significant drop in signal intensity for up to six sorption cycles.

Some studies reported the application of graphitic carbon nitride as the base for a novel dSPE sorbent. Mesoporous graphitic carbon nitride (MCN) was used in the enrichment of sulfonamides in a column-assisted extraction procedure (CA-dSPE) designed for easier separation of the dispersed sorbent [45]. A series of extraction procedures, namely SPE, dSPE and pipette-tipped SPE (PT-SPE), using the MCN sorbent, was carried out for reference; however, none of the standard methods yielded better or even comparable sulfonamide extraction results than CA-dSPE. The proposed method utilizing HPLC-DAD analysis showed good sensitivity and recovery while also requiring very little solvent for analysis when compared to the reference data, and the sorbent itself also showed good reusability. A method for phenoxy carboxylic acid (PCA) compound screening was developed with the use of “velvet-like” graphitic carbon nitride (V-g-C_3_N_4_) as a sorbent [46]. The employment of the direct analysis in real time (DART) ionization technique coupled with an MS detector resulted in achieving remarkable sensitivity, much higher than in both the comparative HPLC-UVD analysis and the cited literature, while requiring application of as little as 1 mg of the sorbent. Considering the fact that the material preparation is not complicated, this method’s biggest drawback appears to be its relatively low extraction accuracy and the complexity and cost of the DART apparatus.

### 5.2. Carbon Nanotubes

Carbon nanotubes (CNTs) are an allotropic form of carbon related to fullerene and graphene. Similarly to fullerene, they form three-dimensional structures composed of carbon atoms ordered in a hexagonal grid; however, they form tubules instead of cages. Single-wall carbon nanotubes (SWCNTs) can be visually described as a graphene sheet rolled into a tube, and multi-wall carbon nanotubes (MWCNTs) consist of multiple SWCNTs nested one over another.

Recent years have seen a significant rise in the importance of these materials, as their remarkable and unique electrical, mechanical and chemical properties have garnered them much attention. CNTs show good thermal and chemical stability, which, combined with their high adsorption capacity, ability to enter into π-π interactions with aromatic compounds as well as the relative ease with which they can be modified, opens up exciting possibilities for their application. While they have already found wide use in other separation techniques, such as standard SPE or SBSE, only recently have carbon nanotubes begun to gain popularity as sorbents in dSPE procedures [4,47,48]. A series of works published by Paszkiewicz et al. describe the performance of several MWCNT types as dSPE sorbents in analytical procedures determining different analytes, including PAHs [4,47], heavy metals [4] and various pharmaceuticals [48,49]. Theoretical and analytical tests saw studies on both modified MWCNTs and unmodified tubes of varied shapes, lengths and diameters. Great results obtained even by coupling the dSPE technique with standard procedures, such as GC-MS or LC-MS (Table 4), show the very high potential of MWCNTs as sorbent materials. Their flexibility in this role was perfectly highlighted in a separate study, where modified MWCNTs intercalated with magnetite nanoparticles were used together with CTAB surfactant to collect gaseous PAHs from marine diesel engine emissions [50]. All sixteen investigated PAHs were captured in the aqueous solution, including the heaviest ones, such as chrysene and indeno(1,2,3-cd)pyrene, and extracted using OH-MMWCNTs@CTAB, with satisfactory recovery, which allowed for detection with high sensitivity using the GC-MS system.

Recently, MWCNTs have also been investigated as a component for “bucky gels”, a novel type of material combining the properties of ionic liquids and nanomaterials. Utilizing carbon nanotubes as a nano component is a way of inhibiting their aggregation while also increasing their dispersibility in water. Such a material can be applied as a sorbent in the dSPE procedure, as was reported in a paper focusing on chromium determination in water [51]. In a follow-up study, the development of a magnetic bucky gel (MBG) based on choline chloride, a hydrophilic deep eutectic solvent (DES), is described, again using MWCNTs [52]. The obtained material was applied as a dSPE sorbent in the determination of chlorine pesticides utilizing a GC-MECD system. Comparison with the reference reveals the remarkable sensitivity of the proposed method, better than in any of the cited studies. Its additional benefits include the exceptionally fast extraction process, which is reported to only take few seconds, as well as the excellent target enrichment (EF values found within 305–335 range), with the relatively wide recovery range appearing as the only notable disadvantage.

**Table 4:** Carbon-based sorbents.

Material	Target Analyte	Sample Matrix	Linear Range (µg L^−1^)	Sensitivity ^a^ (ng L^−1^)	Recoveries (%)	Detection Method	Ref.
AMM-AC	Cu	food	0.8–180	310	95.7–103.6	FAAS	[38]
AC-PAN	antibiotics	waste	-	50–200	98–103	HPLC-DAD	[35]
ACF	pesticides	food	-	1.14–5.89 ^b^	70–120	GC-ECD, GC-MS/MS	[53]
GO@SiO_2_	phytohormones	food	0.5–50	30,000–50,000	98.1–118.4	HPLC-UVD	[39]
GO@SiO_2_	melatonin, tryptophan	food	0.25–500	50,000–100,000	89.1–114.8	HPLC-DAD, UPLC-MS/MS	[36]
3D-Mag-CMGO	disperse dyes	waste	5–1000	500–2480	70–109	UFLC-MS/MS	[41]
3D-Mag-CMGO	disperse dyes	environmental	0.005–5	0.17–10.2	80.0–112	UFLC-MS/MS	[42]
3D-Mag-CMGO	pharmaceuticals	environmental	0.001–0.5	0.034–0.63	78.0–109	UFLC-MS/MS	[43]
tri-BuA-rGO	pesticides	food	1–500 ^b^	0.33–16.5 ^b^	72.1–120.5	UHPLC-MS/MS, GC-MS/MS	[40]
rGO-CHNPs	velpatasvir	biological	0.5–45	40	97.96–103.0	CPE-FLD	[44]
G/Sep	ryboflavin	food	80–700	3000	95–104	FLD	[54]
G/Sep	PAH	waste	0.39–45	96–830	95.2–100.2	HPLC-FLD	[37]
MCN	sulfonamides	environmental	0.09–200	20–50	82.3–110.5	HPLC-DAD	[45]
V-g-C_3_N_4_	PCA	environmental	-	0.5–2	80.12–119.17	DART-MS, HPLC-UVD	[46]
MWCNTs	PAH, Cd, Cr, Pb	environmental	0.01–50	3–30	80.7–116.1	GC-MS/AAS	[4]
MWCNTs	PAH	environmental	-	-	-	GC-MS	[47]
MWCNTs	pharmaceuticals	environmental	0.02–2.5	1–8	85.99–120.05	LC-MS/MS	[48]
MWCNTs	β-blockers	environmental	0.005–0.5	1	80.2–135.7	GC-MS, LC-MS/MS	[49]
QA-Mag-CCNTs	perchlorate	biological	0.01–1 ^b^	0.00249 ^b^	85.2–107	UFLC-MS/MS	[55]
PEG-CNT-MNP	*Z*-ligustilide	herbal	-	-	98.9	HPLC-DAD, HPLC-MS/MS	[56]
M-BG	Cr	environmental	0.4–40	100	94.4–106	FO-LADS	[51]
DES-MBG	pesticides	environmental	0.0002–2	0.03–0.27	80–119	GC-MECD	[52]
OH-MMWCNTs @CTAB	PAH	engine exhaust	0.02–1	9–100	72.65–96.54	GC-MS	[50]

^a^ LOD values; ^b^ µg kg^−1^; ACF: activated carbon fibers; CMGO: magnetite-graphene oxide composite; tri-BuA: tri-butylamine; Sep: sepiolite; PCA: phenoxy carboxylic acid; QA-Mag-CCNTs: quaternary ammonium modified magnetic carboxylic carbon nanotubes; FO-LADS: fiber optic linear array detection spectrophotometry; CTAB: cetylthrimethylammonium bromide.

## 6. Sorbents Based on Layered Double Hydroxides

Layered double hydroxides (LDHs) belong to a class of synthetic anionic clays formed by stacked layers of double hydroxides of divalent and trivalent cations with hydrated anions dispersed between them. A unique property of these materials compared to other sorbent types is their high solubility in acid, which led to the development of extraction methods in which the desorption step is replaced entirely by LDH-based sorbent dissolution, thus reducing the expenditure of organic solvents. An additional benefit of replacing desorption in the analytical procedure is minimizing analyte loss, leading to highly accurate recovery [57,58].

In recent years, there have been several reports on studies investigating LDH materials as dSPE sorbents in analytical procedures aimed at the determination of heavy metals, such as cadmium, cobalt or lead. The accuracy of the extraction process was as high as expected and, in addition, most LDHs showed very good selectivity as well, even when tested with as many as 20 potentially interfering inorganic ions [59]. Many of the studies also report the sensitivity of the proposed methods to be comparable to or better than in the cited literature; however, the provided results are noticeably worse than in other papers mentioned in this review, particularly those using silica or carbon-based sorbents (Table 5).

While most of the studies employed a FAAS detection system, an attempt at chromium(VI) determination with fluorescence, using Mg-Al mixed hydroxide as the dSPE sorbent, was reported [60]. The method showed good selectivity; however, the fluorescence detection sensitivity was poor, even when using a novel probe, while also being applicable in only a narrow concentration range.

**Table 5:** Layered double hydroxide-based sorbents.

Material	Target Analyte	Sample Matrix	Linear Range (µg L^−1^)	Sensitivity ^a^ (ng L^−1^)	Recoveries (%)	Detection Method	Ref.
BCS-LDH	Fe	food, environmental	0.5–100	400	99.04–102.3	UV/Vis	[59]
Mg-Al-AHDNA-LDH	Cd, Co, Cr, Ni, Pb	biological	2–725	600–2400	95–102	FAAS	[61]
LDH-APDC	Cr	biological	8–640	2400	96–101	FAAS	[57]
LDH-ALA	Cr	environmental	20–700	7100	97.67–110.08	FAAS	[58]
DAMP-CuNCs	Cr	environmental	116–812	36,000	101.6–106.9	FLD	[60]

^a^ LOD values; BCS: bathocuproine disulfonic acid; AHDNA: 4-amino-5-hydroxyl-2,7-naphthalendisulfonic acid; APDC: ammonium pyrrolidine dithiocarbamate; ALA: L-alanine; DAMP-CuNCs: 4,6-diamino-2-mercaptopyrimidine-coated copper nanoclusters.

## 7. Sorbents Based on Metallic Organic Frameworks

Porous coordination networks, also known as metallic organic frameworks (MOFs), are crystalline organic–inorganic hybrid materials, formed by metal ions or clusters and organic bridging ligands. Their most notable properties, including high specific surface area, porosity easily tuneable by linker choice and the fact that they are readily modifiable, brought significant attention towards a wide spectrum of industrial and scientific applications. They have already found use in catalysis, drug delivery and energy storage, as well as separation techniques [62,63,64].

One of the more common types of MOF researched recently appears to be zeolitic imidazole frameworks (ZIF), networks based on various metals with imidazolate ligands. A zinc-histamine-based ZIF-8 material showed remarkable efficiency in organophosphorus pesticide extraction [64], as enrichment factors calculated for the analytes were found between 801 and 914, and it retained its high effectiveness for up to eight sorption cycles. Analyte determination was carried out using a gas chromatograph coupled with a flame ionization detector (GC-FID) system, and the proposed method allowed for sensitivity better than in any study cited for reference; however, the narrow applicable concentration range is a noticeable disadvantage (Table 6). In another study, Ghani proposed using a ZIF based on cobalt (ZIF-67) as a precursor in the dSPE procedure with hierarchical Ni-Co LDH (HLDH) sorbent forming in situ [65]. The method additionally retains the benefits of LDH sorbents as HLDH is easily dispersed in 8% trifluoroacetic acid, thus reducing solvent use and analyte loss, resulting in high accuracy. The method proved to be more effective at bisphenol A (BPA) extraction than dSPE with a standard Ni-Co LDH sorbent performed for comparison and also showed good sensitivity when coupled with a HPLC-UVD system.

A copper-based MOF utilizing benzene-1,3,5-tricarboxylic (BTC) moieties as ligands was used as a precursor for a material designed to combine the properties of metallic organic frameworks and graphitic carbon [62]. The final product was characterized as porous octahedron graphitic carbon cages with metallic copper and applied as a dSPE sorbent for the extraction of fluoroquinolones, achieving good target enrichment. Comprehensive selectivity tests showed high tolerance in the presence of glucose, fructose and vitamins, as well as Fe^3+^, Cu^2+^, Ca^2+^, K^+^, Cl^−^ and NO_3_^−^ ions; however, the sorbent retained only 80% of its sorption effectiveness after just four extraction cycles. Comparing the analytical performance of the proposed dSPE-HPLC-UV method with literature data shows exceptional sensitivity, greatly exceeding values reported in any of the cited studies.

## 8. Sorbents Based on Porous Polymers

Porous polymers based on organic crosslinked resins form beads of uniform diameter with regular pore sizes. The properties of the material depend on the monomers used and the parameters of the polymerization reaction, allowing for high control over the final product and reliability of the process. Their advantages over other porous materials include inherent functional diversity and physicochemical stability, especially against water, while exhibiting very high surface areas. These remarkable properties granted polymers wide application in catalysis, gas storage and photoluminescence, among others, and their structural uniformity makes porous polymers excellent packing materials, commonly used in column-based separation techniques [1,70,71].

Recently, there has been also a growing interest in investigating their applicability in the dSPE technique (Table 7), including a study focusing on a series of materials using poly(styrene-divinylbenzene) (PS-DVB) copolymer or silica cores modified with N-vinyl pyrrolidon (NVP), 1H,1H,7H-dodecafluoroheptyl methacrylate (DMFA) or both [6]. All of the obtained materials were investigated as sorbents for flavonoid extraction. P-N-F (PS-DVB-NVP-DMFA) showed the highest sorption capacity of both quercetin and pyrocatechin and was therefore selected for further study. When combined with the HPLC-UV/Vis detection system, the procedure yielded very good sensitivity; however, reported target recoveries were relatively low.

The potential for porous polymers as sorbents of choice is illustrated by research focusing on polyethyleneimine (PEI), a weak polymeric anion exchanger, in a direct comparison with PSA regarding applicability for the simultaneous extraction of multiple pesticides and matrix clean-up of QuEChERS extracts [71]. In most cases, PEI performance appears to be similar or better, including much more accurate target recoveries as well as more effective fatty acid clean-up. In addition, a calculation of material cost per sample suggests PSA to be nearly five times more expensive to use than PEI.

## 9. Other Novel Sorbents of Note

Various materials have been thoroughly researched as sorbents applicable to the dSPE procedure that do not necessarily belong to any of the types more commonly investigated. One such material is molybdenum disulfide (MoS_2_), a transition metal dichalcegonide forming two-dimensional sheets of sulfide atoms with molybdenum trapped between them. Reports describing the application of MoS_2_ for sulfonamide determination in the dSPE procedure coupled with capillary zone electrophoresis (CZE) [75,76] yielded somewhat disappointing results, showing low recovery accuracy and sensitivity (Table 8). A composite of molybdenum disulfide and carbon dot (MoS_2_/CD) used to extract flame retardants [77] produced results much closer to data reported in referential studies and also substantially exceeded the performance of unmodified MoS_2_ and CD. The composite was also shown to be reusable for up to seven extraction cycles.

As metal oxides are known to exhibit high selectively towards cis-diol moieties, reports describe medical studies focusing on the determination of ribose conjugates in biological fluids that employ a dSPE clean-up step with CeO_2_ as a sorbent [78,79]. The selectivity of the sorbent was confirmed by performing the extraction of four standard ribonucleosides from a solution of potentially interfering 2′-deoxynucleosides; however, target recoveries from real samples were low. Additionally, labeling analytes with a pair of stable isotope labeling (SIL) reagents significantly improved the sensitivity of the HPLC-MS/MS detection system, allowing for successful identification of 50 potential ribose conjugates in each study.

## 10. Summary and Conclusions

The necessity to determine trace amounts of numerous xenobiotics present in environmental samples containing varied and complex matrixes commands the drive to search for increasingly sensitive and selective methods of detection, as well as pre-treatment procedures that diminish the negative sample matrix effect. Among many known and widespread extraction methods used for analyte concentration prior to qualitative or quantitative analysis, dispersive solid phase extraction is a relatively new development of the classic SPE technique. In recent years, a significant increase in interest in dSPE can be observed, as this remarkably simple, rapid and effective technique finds use in a growing number of applications.

Articles surveyed for the purpose of this review presented studies on a wide variety of samples ranging from environmental soil and water samples to fresh produce, grain, herbs and meat, to processed food products, to biological and medical samples as well as pharmaceutical products. The reach of the analyzed xenobiotics was similarly broad, including heavy metal and rare earth metal ions, various pesticides, pharmaceuticals and their metabolites, PAHs, dyes, flame retardants and others. Procedures described in these studies were also varied in their utility, with some methods designed for the highly selective extraction and detection of a particular chemical, while others were refined to carry out a simultaneous determination of dozens of compounds of a specific type. This proves the high adaptability of the dSPE technique and suggests that the scope of possible applications for which this method is suitable will further increase in the future.

Nanomaterials based on various carbon allotropes, such as graphene or carbon nanotubes, are currently the most popular research subject, with only recently over twenty papers published. Several other sorbent types were also broadly investigated as suitable for dSPE—for instance, standard and mesoporous silica, magnetite-based nanoparticles, metallic organic frameworks and others.

There is a strong trend of modifying previously known, classical sorbents to acquire hybrid materials. The goal is to create sorbents combining the properties of two or more different materials such as carbon-based nanoparticles, MNPs and polymers. This multifunctional sorbent could then be applied for the extraction of multiple analytes; thus, it is essential to ensure that it retains most of the effectiveness of its parent materials. In parallel, a lot of attention is given to highly specialized sorbents such as MIPs, focusing on extracting particular compounds with extremely high efficiency. As research further refines and improves sorbent properties, focusing on extracting either multiple targets from complex matrixes or a selected few analytes with very high efficiency appear to be the two most prominent paths for the application of dSPE as a pre-treatment technique in the future.

What needs highlighting is that, in the majority of the presented studies, procedures utilizing these novel sorbents in dSPE produced very good results. In some cases, the precision and sensitivity of the analysis competed with the most accurate methods reported to date, while most have traded some small measure of analytical performance for the remarkable operating simplicity, greatly shortened process time and lower solvent, as well as sorbent and sample amount requirements. This implies that the popularity of dSPE as a standard sample pre-treatment procedure should grow, especially in large, high-throughput laboratories dealing with large amounts of samples, where decreased cost and analysis time would matter most, since the trade-off in analytical performance is often minimal, if any at all.

At the same time, as more sorbents and improvements to the technique itself are developed, its advantages over other extraction procedures may very well outweigh any negatives.

## Figures and Tables

**Table 1:** Silica-based sorbents.

Material	Analyte	Sample Matrix	Linear Range (µg L^−1^)	Sensitivity ^a^ (ng L^−1^)	Recoveries (%)	Detection Method	Ref.
SiO_2_-BPHA	rare earth elements	aqueous solution	-	-	-	ICP-OES	[12]
NIPAAm-*co*-ABTA_0.35_ @SiO_2_	α-casein	food	-	-	-	HPLC-DAD	[13]
LMA-HEDA@SiO_2_	herbicides	environmental	0.1–4	27–53	80.1–97.9	HPLC-UVD	[14]
QTA-MCM-48	EDC	environmental	0.005–0.5	1.2–2.6	95.4–104	HPLC-FLD	[15]
HMS-RPC8-SAX2	polyphenols	food	-	1–560	70–101	UHPLC-MS/MS	[9]
HMS-C18	polyphenols	food	0.02–100	10–50	48–103	UHPLC-MS/MS	[16]
SBA-15/Met	Cd, Ni, Pb	food, environmental	0.0025–10	1–2	97.9–101.5	GFAAS	[10]
SBA-15/CCMet	Cd, Pb	food, environmental	0.001–15	0.2, 0.5	96.4–101.9	GFAAS	[17]
MSN-NH_2_	synthetic dyes	food	0.45–1000	0.10, 0.30	80.0–116.8	HPLC-DAD	[11]
IL-WFOMS	plant growth regulators	herbal	0.05–22.5 ^b^	0.003–0.008 ^b^	77.6–98.3	HPLC-FLD	[8]

^a^ LOD (limits of detection) values; ^b^ µg kg^−1^; BPHA: *N*-benzoyl-*N*-phenylhydroxylamine; ICP-OES: inductively coupled plasma optical emission spectrometry; NIPAAm-*co*-ABTA_0.35_: poly[(*N*-isopropylacrylamine-*co*-4- (3-acryloythioureido) benzoic acid)_0.35_]; LMA-HEDA: poly(lauryl methacrylate-*co*-1,6-hexanediol ethoxylate diacrylate); QTA-MCM-48: quaternary ammonium-Mobil Composition of Matter-48; HMS-RPC8-SAX2: Hexagonal Mesoporous Silica dual-functionalized with n-octyl and anion exchange groups; SBA-15/Met: Santa Barbara Amorphous-15/metformin; GFAAS: graphite furnace atomic absorption spectrometry; CCMet: cyanoric chloride-metformin; IL-WFOMS: ionic liquid-functionalized ordered mesoporous silica.

**Table 2:** Magnetic nanoparticle-based sorbents.

Material	Target Analyte	Sample Matrix	Linear Range (µg L^−1^)	Sensitivity ^a^ (ng L^−1^)	Recoveries (%)	Detection Method	Ref.
GO-Fe_3_O_4_	tamsulosin hydrochloride	biological	0.5–50	170	98.0–101.4	HPLC-UVD	[18]
Fe_3_O_4_@pDA	mycotoxins	food	20–400	290–4800, 0.41–5.82 ^b^	77–120	HPLC-MS/MS	[20]
Fe_3_O_4_@pDA	phthalic acid esters	environmental	0.5–500	9–20 ^c^	71–120	GC-MS/MS	[21]
Fe_3_O_4_@pDA	phthalic acid esters	environmental	0.1–250	1.38–3.19 ^c^ 0.020–4.0 ^b c^	70–120	GC-MS/MS	[22]
M-PMA	As	environmental	0–100	2.98–9.95	99–102	HG-MP-AES	[23]
Zein@Fe_3_O_4_	trihalomethanes	environmental	0.5–100	100–360	96.68–101.2	GC-MECD	[24]
Fe_3_O_4_@PVA	antibiotics	food	20–4000 ^b^	0.913–1.23 ^b^	82.9–100.7	HILIC-MS/MS	[25]
SAC-MNP	Pb	food	30–250	10,000	102.6–106.6	SQT-FAAS	[26]
SAC-MNP	EDC	environmental	1–1000	0.28–10,000	95.3–107.8	GC-MS	[27]

^a^ LOD values; ^b^ µg kg^−1^; ^c^ LOQ (limits of quantification) values; GO: graphene oxide; pDA: poly(dopamine); PVA: poly(vinyl alcohol); HILIC: hydrophilic interaction liquid chromatography; SAC: stearic acid coating; SQT-FAAS: slotted quartz tube flame atomic absorption spectrometry.

**Table 3:** Molecularly imprinted polymer-based sorbents.

Material	Target Analyte	Sample Matrix	Linear Range (µg L^−1^)	Sensitivity ^a^ (ng L^−1^)	Recoveries (%)	Detection Method	Ref.
Fe_3_O_4_@Cr(VI)IIPs	Cr	environmental	4–140	29,000	96.1–99.2	FAAS	[19]
Pb-IIP	Pb	food	3–900	700	96.0–104.0	FAAS	[30]
Ag-IIP	Ag	environmental	0.5–600	90	96.2–105.7	FAAS	[31]
MMIP	PAH	environmental	0.002–50	1–100	4.5–97	GC-MS/MS	[28]
GO-MIP	cefadroxil	environmental	40–6000	10,000	72.5–104.8	UPLC-DAD	[32]
dt-MIP	fluoroquinolones	environmental	1–200	220, 360	80.9–101.0	HPLC-DAD	[33]
HMIM	azoxystrobin	food	100–10,000 ^b^	0.324 ^b^	85.93–88.89	HPLC-UVD	[29]
PD-MMIP	PD, resveratrol	medicine	10–10,000	2500, 3500	91.8–102.2	HPLC-DAD	[34]

^a^ LOD values; ^b^ µg kg^−1^; IIP: ion imprinted polymer; MMIP: magnetic molecularly imprinted polymer; PD: polydatin.

**Table 6:** Metallic organic framework-based sorbents.

Material	Target Analyte	Sample Matrix	Linear Range (µg L^−1^)	Sensitivity ^a^ (µg L^−1^)	Recoveries (%)	Detection Method	Ref.
Cu@graphitic carbon cages	fluoroquinolones	food, environmental	0.1–500 1–500 ^b^	0.018–0.042 0.61–1.76 ^b^	81.3–104.3	HPLC-UVD	[62]
carboxylated ZIF-8	methamphetamine	biological	50–2500	10	99.83	HPLC-UVD	[63]
zinc-based MOF	pesticides	environmental	0.1–100	0.03–0.21	91.9–99.5	GC-FID	[64]
HLDH	bisphenol A	environmental	0.5–200	0.12	92–97	HPLC-UVD	[65]
NH_2_-MIL-101	bisphenols	environmental	0.05–200	0.016–0.131	90.8–117.8	HPLC-FLD	[66]
UiO-66	insecticides	environmental	10–500	0.02–0.4	73.7–119.0	HPLC-MS/MS	[67]
Fe_3_O_4_@Fe-BTC	blood lipid regulators	environmental	585–15,400	170–467	86.7–99	HPLC-UV/Vis	[68]
MOF-5	thiols	environmental	0.118–276	0.0016–0.0031	86.6–98.5	HPLC-FLD	[69]

^a^ LOD values; ^b^ µg kg^−1^; MIL: Material Institute Lavoisier; UiO: Universitete I Oslo.

**Table 7:** Polymer-based sorbents.

Material	Target Analyte	Sample Matrix	Linear Range (mg L^−1^)	Sensitivity ^a^ (µg L^−1^)	Recoveries (%)	Detection Method	Ref.
PEI	pesticides	food	-	-	91–105	TLC, LC-MS	[71]
PANI-NaY	pesticides	food, environmental	0.05–50	1–310	64–128	HPLC-DAD	[72]
dPPA	food colorants	food	100–50,000 ^b^	0.035–0.055 ^b^	94.3–102	FASI-CE-C^4^D	[73]
CDP	quinolones	environmental	0.025–5	2.67–5.50	95.47–103.3	HPLC-UVD	[74]
P-N, P-N-F, Si-N, Si-N-F	pyrocatechin, quercetin	food	1–400	50, 80	78.06–83.63	HPLC-UV/Vis	[6]

^a^ LOD values; ^b^ µg kg^−1^; TLC: thin layer chromatography; PANI-NaY: NaY zeolite coated with polyaniline; dPPA: dispersive powder polyamide; FASI-CE-C^4^D: capacitively filed amplified sample injection capillary electrophoresis-coupled contactless conductivity detector; CDP: cyclodextrin-based polymer.

**Table 8:** Other notable sorbents.

Material	Target Analyte	Sample Matrix	Linear Range (µg L^−1^)	Sensitivity ^a^ (µg L^−1^)	Recoveries (%)	Detection Method	Ref.
PEG@MoS_2_	sulfonamides	food	300–30,000	30–200	61.80–110.91	CZE-DAD	[75]
MoS_2_	sulfonamides	environmental	500–50,000	50–120	73.20–111.51	CZE-DAD	[76]
MoS_2_/CD	flame retardants	environmental	1–100	0.01–0.06	80–91	HPLC	[77]
CeO_2,_ ZrO_2_	ribose conjugates	biological	-	0.16–1.59 ^b^	78.5–97.5	HPLC-MS/MS	[78]
CeO_2_	ribose conjugates	biological	-	4.11–18.09 ^b^	34.0–55.9	UHPLC-MS/MS	[79]
[C_16_MIM]Br-AL	insecticides	environmental	1–500	0.14–0.21	70.6–97.8	HPLC-DAD, HPLC-UVD	[80]
[C_12_MIM]Br-ATP	pyrethroids	environmental	2–500	0.3–0.6	90.28–107.56	HPLC-DAD	[81]
TFA-TAPB	NAC	environmental	100–50,000	30–90	84.0–112.3	HPLC-DAD	[82]
N-Mag-COF	disperse dyes	textile	0.5–200 ^c^	0.021–0.058 ^c^	72.2–107	UFLC-MS/MS	[83]

^a^ LOD values; ^b^ pg L^−1^; ^c^ µg kg^−1^; [C_16_MIM]Br: 1-hexadecyl-3-methylimidazolium bromide; AL: alkalized luffa sponge fibers; [C_12_MIM]Br: 1-dodecyl-3-methylimidazolium bromide; ATP: attapulgite; TFA: 2,3,5,6-tetrafluoroterephthalaldehyde; TAPB: 1,3,5-tris(4-aminophenyl)benzene; NAC: nitroaromatic compounds; N-Mag-COF: N-doped magnetite-covalent organic framework composite.
